# The impact of confirmed cases of COVID-19 on residents’ traditional Chinese medicine health literacy: A survey from Gansu Province of China

**DOI:** 10.1371/journal.pone.0285744

**Published:** 2023-11-14

**Authors:** Ge Zhu, Xiulan Wang, Tengyue Zhang, Wangping Zhao, Li Ma

**Affiliations:** 1 School of Economics, Trade and Management, Gansu University of Chinese Medicine, Lanzhou, Gansu, China; 2 School of Public Health, Gansu University of Chinese Medicine, Lanzhou, Gansu, China; 3 School of Traditional Chinese and Western Medicine, Gansu University of Chinese Medicine, Lanzhou, Gansu, China; Beijing University of Chinese Medicine, CHINA

## Abstract

Since the outbreak of the new crown epidemic in China in early 2020, the number of confirmed cases of COVID-19 has continued to increase, and the Chinese government’s policy of "static management" in the first round of the epidemic may affect the health behavior adjustment of Chinese residents. Using survey data on the TCM health literacy of 4016 residents in China (Gansu Province), a causal inference approach was used to explore the impact of the emergence of confirmed cases of COVID-19 on residents’ TCM health literacy. We found that the emergence of confirmed cases can increase by 3.5%-7.0% in residents’ TCM health literacy. Among them, the TCM health literacy of uneducated residents has not improved significantly, and the residents with secondary education have increased significantly by 8%. For those with higher education, the number of residents increased significantly by 6%. At the same time, the emergence of confirmed cases will increase the residents’ practical TCM health literacy and decrease theoretical TCM health literacy. Through heterogeneity analysis, we explored the impact mechanism of confirmed cases on residents’ TCM health literacy. We believe that the emergence of confirmed cases will make residents more inclined to participate in TCM-free clinics, theme activities, and other ways to acquire TCM knowledge.

## Introduction

COVID-19 is a global public health event that countries including China are experiencing. The full-scale outbreak of COVID-19, which compels residents to obtain, identify and apply health information, has emphasized that health literacy is an underestimated global public health issue [1]. In Europe, for example, nearly half of adults’ report having health literacy issues and lack the associated ability to take care of their health and that of others. Health literacy has been recognized as an important tool for the prevention of non-communicable diseases, and government education and dissemination of health literacy have taken sustainable long-term measures from early in the life course of residents [2]. However, when COVID-19 quickly emerged, two aspects became noticeable. First, on a global scale, health literacy is equally important for preventing communicable diseases as it is for preventing non-communicable diseases. Second, in addition to social medical system preparation, individual preparation is also key to solving complex real-world problems. In this pandemic, improving the health literacy of residents in a short period is difficult, but possible, as governments and citizens need to take immediate action due to the emergence of locally confirmed cases [3, 4].

In China, the health literacy of Chinese residents differs from that of most other countries. The Chinese government has preserved the time-honored traditional medicine, so China’s medical and health system is based on both Western medicine and traditional Chinese medicine, which is a fully institutionalized and government-supported part of the Chinese medical system. Western medicine has the same legal status. It provides nearly 40% of healthcare services in contemporary China [5]. Therefore, the health literacy of Chinese residents should include the part of traditional Chinese medicine, that is, health literacy of traditional Chinese medicine. If someone does not consider the health literacy of Chinese medicine residents when summarizing the health literacy of Chinese residents, we think this is unreasonable and cannot represent the overall Chinese residents. Health literacy level, Chinese medicine health literacy of Chinese residents is often overlooked, and Chinese medicine has played an important role in dealing with the COVID-19 incident. Therefore, it is necessary to study the health literacy of traditional medicine, at least for Chinese residents [6]. With the help of the COVID-19 pandemic, we will focus on exploring whether the occurrence of COVID-19 infection in a region affects the health literacy of residents in traditional Chinese medicine, that is, whether the disclosure of information about confirmed cases will affect the health behavior of residents.

In this study, we conducted a TCM health literacy monitoring survey on 4,016 residents from Gansu Province before and after the epidemic, which included respondents’ exposure to TCM, knowledge of TCM, basic knowledge of TCM, and Basic information about the respondents themselves and their families. We focused on monitoring eight areas, which showed different severity of the epidemic after 2020. Some areas did not have confirmed residents after the outbreak of COVID-19, while others did. We made three possible assumptions based on factors such as China’s (Gansu) epidemic prevention policy and the traffic in each monitoring area: First, it is assumed that the eight monitoring areas are independent and relatively closed after the outbreak of COVID-19, and the population mobility is poor [7]; Second, if a confirmed case occurs in a certain area, the area should be closed and managed immediately to prevent the possibility of secondary transmission and outward spread of infected persons [8]; third, it is assumed that only residents of areas with confirmed cases are at risk of infection, and residents of areas without confirmed cases have no (or low) risk of infection [9, 10].

Based on the hypothesis of possibility, we can classify our samples twice: first, according to the distinction between samples before and after COVID-19, we use the samples in 2019 as the control group and the samples in 2020 as the control group. The samples are used as the experimental group; the second is to distinguish according to the areas with confirmed cases after the outbreak of COVID-19, residents in areas without confirmed cases are used as the control group, and residents in areas with confirmed cases are used as the experimental group. After identifying the control group and the experimental group, we used the DID (difference-in-difference) method to explore whether the occurrence of confirmed cases had an impact on the residents’ TCM health literacy. In our results, the TCM health literacy of residents in areas with confirmed cases was significantly higher than that in areas without confirmed cases, and this conclusion was confirmed by two placebo tests.

More interestingly, the changes in TCM health literacy also showed hierarchical changes among residents of different socioeconomic statuses. Unfavorable socioeconomic status can lead to low health literacy, especially in terms of educational attainment, which has the greatest impact on residents’ health literacy. So, we divided all residents into groups according to their educational level. We also explored the mechanism at the end. The source of this effect lies in the difference in residents’ immediate sexual response to confirmed cases in the local area, which expands the level of residents’ TCM health literacy.

The main contributions of this study are as follows: First, it confirms the “confirmed case” factor in the improvement of Chinese residents’ TCM health literacy during the COVID-19 pandemic; There are significant differences in the improvement of health literacy; the third is to explore the specific aspects and specific mechanisms of the impact of confirmed cases of COVID-19 on residents’ traditional Chinese medicine health literacy; the fourth is to use economics and epidemic methods to solve problems in the field of public health, which is an important attempt for interdisciplinary integration development.

It and chapters are arranged as follows: The second part briefly introduces the anti-epidemic background and traditional Chinese medicine background of the COVID-19 pandemic in China (Gansu), and discusses our theoretical assumptions in detail; the third part introduces the data sources of this study, variables and descriptive statistics; the fourth part describes the identification strategy, model setting and empirical results of this study; the fifth part is the conclusion.

## Background

China, the first country to suffer from COVID-19, has moved quickly to prevent and control the virus due to its emergency health policy. The introduction of the first-level public health emergency response policy can be traced back to 2007 when the Chinese government accumulated experience in dealing with the severe acute respiratory syndrome (SARS) and formulated the Emergency Response Law of the People’s Republic of China. During the first-level public health emergency, local governments need to demarcate control areas, seal off epidemic areas, take coercive measures (i.e., restrict or suspend work, production, and business), implement restrictions on the movement of people, establish quarantine stations, and collect information from various communities. With the outbreak of COVID-19, the number of newly confirmed cases of infection in China has been increasing, and it has attracted the attention of the central government and the implementation of emergency health policies. China has enacted restrictions on the movement of people.

While ensuring the reduction of future confirmed cases, the Chinese government is also actively treating residents infected with COVID-19. Based on China’s special health system, Chinese medicine has a long history and has been an important part of the Chinese people’s fight against various epidemics since ancient times. means, including the 2003 SARS or the 2019 pandemic. The General Office of the Chinese Health Commission and the China Administration of Traditional Chinese Medicine issued the "Diagnosis and Treatment Plan for Novel Coronavirus Infected Pneumonia" (hereinafter referred to as the "Plan"), which reaffirmed the obvious role of traditional Chinese medicine in the treatment of COVID-19.

The COVID-19 pandemic has forced us to refocus on the health literacy of residents [4]. There is no doubt that the improvement of residents’ health literacy will effectively improve the overall efficiency of solving public health events, and have a significant effect on reducing the infection rate and mortality rate of residents [11–15]. Then, for China, after experiencing the first round of the epidemic, the health literacy of Chinese residents has been improved, which can play a positive role in coping with repeated epidemics in the future, and due to the particularity of China’s medical and health system and the data of this study, we It was decided to look for the causes of COVID-19 from the changes in traditional Chinese medicine health literacy among Gansu residents.

To prevent the continued expansion of COVID-19, in addition to the factors of behavior adjustment caused by the improvement of residents’ health literacy, the important factor is the government’s intervention policy. Some empirical studies by scholars around the world pay close attention to the government’s public health emergency. role in incident emergency management, as the government has the primary responsibility for governance in such situations. In fact, since the emergence of COVID-19, the early warning, monitoring, and response to the crisis have all been inseparable from government actions [16–21]. These studies generally agree that the public’s compliance with government-recommended protective behaviors is critical to controlling the spread of epidemics [22]. Therefore, during the COVID-19 pandemic, the adjustment of public health behavior is an important research topic. Such studies also generally target residents who have experienced public health events and regard the changes in residents’ health literacy as the key to residents’ behavior adjustment. Important reasons [23].

We speculate that in this case, the occurrence of confirmed cases of COVID-19 in the living area of the residents is the greatest impact and effect of the residents’ behavior adjustment (changes in health literacy) on the residents, so we have made possible this impact. Sex Assumptions:

First, assuming that the areas where residents live are relatively independent, when COVID-19 cases occur in areas beyond the living areas, this independence effect will be enhanced, making population mobility less mobile. The Chinese government issued a nationwide announcement when there were a large number of COVID-19 cases in Wuhan: Beware of foreigners bringing the virus into local jurisdictions. Local governments have also asked residents to "do not leave their territories unless necessary, and do a good job of home isolation." At the beginning of the COVID-19 outbreak, China was about to enter the "Spring Festival" stage. At this time, due to the pandemic, the "Spring Festival" was suspended, making most of the flow The population stays in place, and some residents choose to "Chinese New Year" in other places. Gansu Province itself is located in northwest China, with mountains and ravines, relatively inconvenient transportation, and a large labor exporting province, which made Gansu Province suffer from poor population mobility and relatively fixed and independent living areas during the first round of the epidemic. The first round of the epidemic lasted only a month [24, 25].

Second, assuming a confirmed case occurs, closure measures should be taken promptly, and the government can successfully eliminate the possibility of secondary transmission of infected people and the spread of the virus to other areas. Both the central government and the local government implemented "static management" measures in the early stage of the epidemic, that is, "one place is closed for one case". Both facts and studies have demonstrated that this policy has a positive effect on suppressing the spread and spread of COVID-19 while sacrificing related economic benefits [26–29].

Third, assuming that only confirmed cases occur within residents’ living areas, residents will have sufficient reasons to adjust their behavior to avoid being infected. If the first two hypotheses are justified, we believe that the third hypothesis should also be justified. Residents’ risk awareness includes three aspects: the perceived severity of COVID-19, the mortality rate of COVID-19, and the degree of impact of COVID-19 on their lives [30–35], at the beginning of the epidemic, Especially when no infected person with COVID-19 is found in the living area of residents, residents do not have a deep understanding of the severity of COVID-19. For Gansu Province, the mortality rate of COVID-19 in Gansu Province was 91 confirmed in the first round of the epidemic, and the last one died. As of October 16, 2022, the cumulative number of confirmed cases in Gansu Province was 1,351, and the cumulative number of cured patients was 1,349. A total of 2 people died, and the mortality rate was 0.148%, which was a low level in China. When there are no new crown cases in the residents’ living areas, the residents’ lives are not greatly affected, but when there are new crown cases in the residents’ living areas, the government will implement "static management" on the local area, and almost all residents are required to isolate at home, so the residents’ Living in this situation will be severely affected. Therefore, during the period of COVID-19, there were no new crown cases in the living area of residents. We can think that residents have a low-risk perception of COVID-19, and few people will adjust their life behavior to deal with COVID-19 with great fanfare [8, 36–39].

To sum up, we put forward the above hypothesis based on the research results of existing scholars, the purpose of which is to construct a theoretical framework that "the occurrence of confirmed cases of COVID-19 in the territory is a determinant of the improvement of residents’ TCM health literacy". The basis for the argumentation of the central thesis by the academic method.

## Date

The sample includes 4,016 permanent residents aged 15–69 from 8 monitored counties (districts) in Gansu Province from 2019 to 2020. 3 townships (streets) are selected from each monitored county (district), and 2 are selected from each township (street). Each household in the village (neighborhood committee) selects one eligible survey object until 45 questionnaires are completed. The questionnaire was conducted using the "Chinese Citizens Chinese Medicine Health and Cultural Literacy Questionnaire" uniformly compiled by the China Administration of Traditional Chinese Medicine. A paper questionnaire was used on-site at households, and the investigators completed face-to-face inquiries. The questionnaire for each resident mainly consists of three parts, the first is the investigation of the popularization of TCM knowledge, the second is the main test of TCM health literacy, and the last is the personal information of the residents (For details about the questionnaire, please refer to the S1 Appendix).

The question explored in this study is whether confirmed cases of COVID-19 can affect residents’ TCM health literacy. We constructed two groups of control samples, one before and one after control, we set the residents surveyed in 2019 as the control group, and the residents surveyed in 2020 as the experimental group, which is the first difference of the DID model; It is the control of whether there have been confirmed cases in the living districts and counties of the residents. We set the residents of the areas with confirmed cases of COVID-19 in 2020 as the experimental group (the 2019 sample also set the residents of these areas as the experimental group). Residents in areas with no confirmed cases of COVID-19 in 2019 were set as the control group (the 2019 sample also set residents in these areas as the control group), which is the second difference of the DID model. As shown in Table 1, a year represents the comparison before and after, and Confirmed cases represent whether there are confirmed cases in the living counties of residents. The variables "Year" and "Confirmed cases" are the explanatory variables of this article, and "Score" is the explained variable of this article. In addition to the above three main variables, in order to reduce the endogeneity of the experiment, we introduced other relevant control variables:

From the perspective of the individual characteristics of the respondents, the control variable "gender" was added, of which male respondents accounted for 48% and female respondents accounted for 52%. The control variable "age" was added, among which 12.5% of the respondents were between 15 and 30 years old; 42.1% were between 31 and 50 years old; Visitors accounted for 45.4%. Added the control variable "education level", a total of 24.8% of the respondents had no schooling; 20.3% of the respondents had a primary school education; 23.3% of the respondents had a junior high school education; 15.5% of the respondents 15.6% of the respondents had a bachelor’s degree; 0.5% of the respondents had a postgraduate degree. The control variable "ethnicity" was added, and the Han nationality accounted for 88.8% of the respondents; the ethnic minorities accounted for 11.2%. We also summarized the occupations of the respondents. 1–9 represent civil servants, teachers, medical personnel, personnel from other public institutions, students, farmers, workers, personnel from other enterprises, and other occupations, accounting for 1.5% of the total, 2.9%, 1.1%, 3.1%, 4.6%, 62%, 5.0%, 5.7%, 14%. The control variable "disease or not" was added, and 55.3% of the respondents suffered from chronic diseases such as hypertension, heart disease, cerebral thrombosis, and diabetes.

From the perspective of the family characteristics of the respondents, adding the control variable "family size", 17.2% of the respondents lived alone; 67.6% of the respondents had 2–4 people in their families; The above respondents accounted for 15.2%. Adding the control variable "household income", about 10.6% of the respondents had an annual household income of less than 10,000 yuan; about 62% of the respondents had a family income between 10,000–49,999 yuan; and their household income was between 50,000–100,000 Respondents whose household income is more than 100,000 yuan accounted for about 20.9%; respondents whose household income was greater than 100,000 yuan accounted for 6.5%.

**Table 1:** Summary statistics.

Variable	Describe	Obs.	Mean	Std. dev.	Min	Max
**Numeric variable**						
Score	Score of traditional Chinese medicine health literacy	4016	51.61504	20.85104	2	96
Age	Respondent’s age	4016	47.29706	13.17334	15	69
Income	Household income of respondents	4016	37009.24	37557.14	0	800000
Population	Respondent’s household population	4016	2.842629	1.627024	1	16
**Classification variable**						
Year	Distinguish between respondents before and after COVID-19	4016	---	---	2019	2020
Con	The confirmed case in the respondent’s residence is 1, not 0	4016	---	---	0	1
Gender	1 male and 2 female respondents	4016	---	---	1	2
Sick	1 for respondents with chronic diseases, otherwise 0	4016	---	---	0	1
Han	if the respondent is Han, otherwise 0	4016	---	---	0	1
Occupation	Occupation of respondents	4016	---	---	1	9
Education	Education level of respondents	4016	---	---	1	6

Note: This table summarizes the demographic characteristics of the residents and their families, as well as the residents’ TCM health literacy scores and other related data. Among them, the number of respondents in 2019 was 1969, and the number of respondents in 2020 was 2047. The respondents in these two years are not exactly the same.

**Fig 1:**
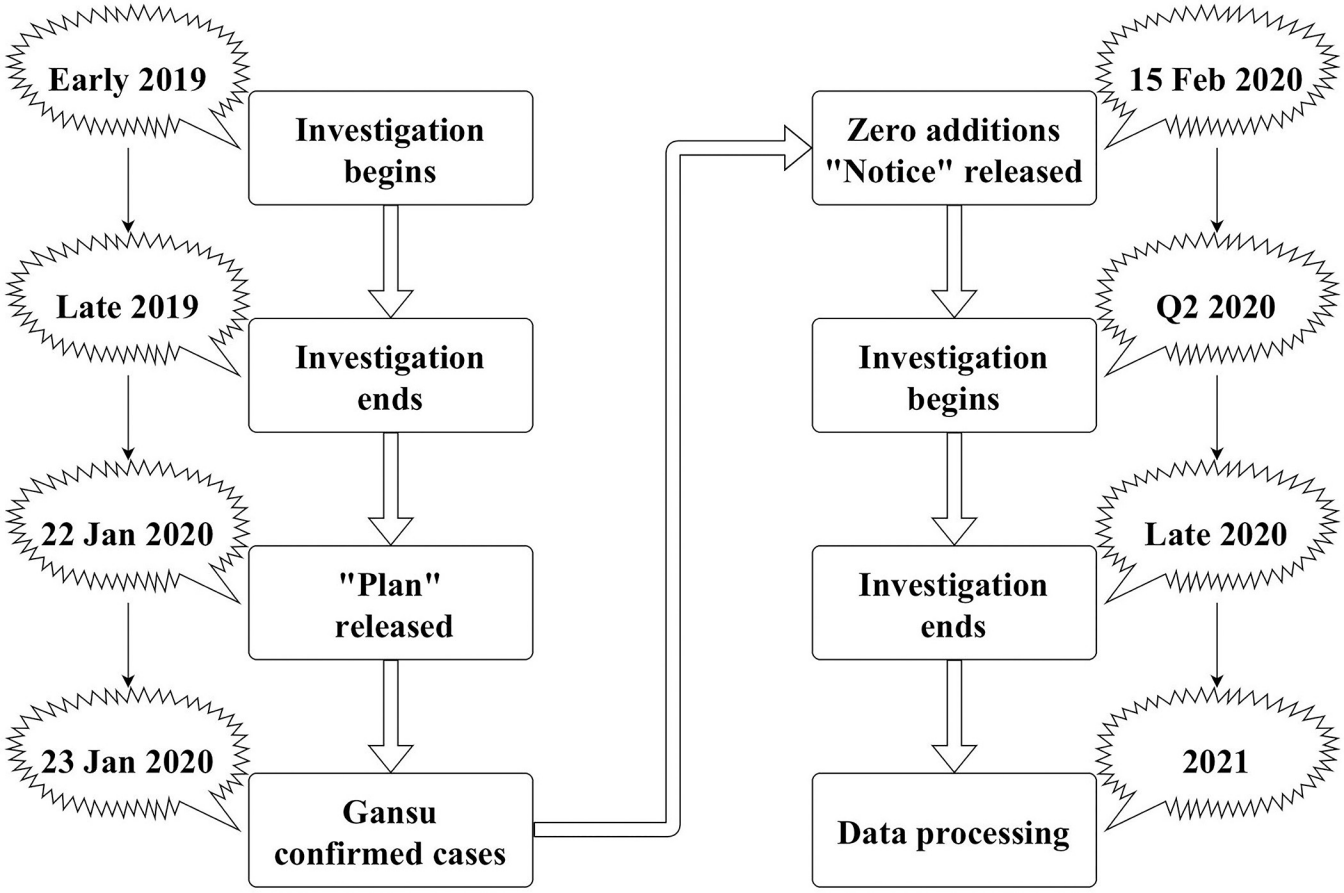
Flow chart of monitoring and investigation period. Note: This picture shows the relationship between the time of the TCM health literacy monitoring survey of residents in Gansu Province and the time of the COVID-19 outbreak. Before the outbreak of COVID-19, we collected data on the TCM health literacy of residents in Gansu Province in 2019. After the first round of the epidemic in Gansu Province ended, we started the 2020 survey on the TCM health literacy of residents in Gansu Province and surveyed the TCM health literacy of residents in Gansu Province in 2020. Complete the monitoring survey before starting.

Two things are worth noting about the findings of this survey. The first is the period of the survey (Fig 1). Regarding the 2019 questionnaires, we have already withdrawn them all before the outbreak, so the respondents can’t predict the arrival of the epidemic and deliberately check the knowledge related to traditional Chinese medicine., Regarding the questionnaire for 2020, we conducted it under the condition that there were no new local cases in Gansu Province after the outbreak, and the survey results were all withdrawn before the next new local case in Gansu Province, so this is A completely independent experiment, the 2019 survey results were identified as pre-COVID-19, and the 2020 survey results were fully identified as post-COVID-19. The second is about the score of the second part of the questionnaire. Regarding the score of the questionnaire, the questionnaires we used before and after COVID-19 are all the same set of questionnaires, without any modification, and there is no need to worry about the difficulty of the test. There is no need to standardize or rank the results of the survey scores due to deviations in the scoring standards of residents before and after COVID-19.

### Identification strategies and empirical results

#### Identification strategy

Before the COVID-19 pandemic occurred, no one could predict the occurrence of a pandemic. COVID-19 is completely an exogenous shock to individuals, so it is a natural experiment with randomness. At the same time, to enhance the effect of COVID-19 on individual behavior, we take confirmed cases in the living area of residents as the standard. We borrow from [40, 41] and use a Difference-in-Difference (DID) model for cross-sectional data:

$${Score}_{its}=\beta_{0}+\beta_{1}{Con}_{is}\times {COVID}_{ts}+\beta_{2}{COVID}_{ts}+\beta_{3}{Con}_{is}+\beta_{4}X_{its}+\varepsilon_{its}$$ (1)

In Eq 1, *Score*_*its*_ is the TCM health literacy test score of resident i in region s in year t, as the explained variable. Among the explanatory variables, *Con*_*is*_×*COVID*_*ts*_ is the interaction item between the occurrence of confirmed cases and the occurrence of epidemics, which is also the main research object of this study. The effect of literacy, *β*_1_ is the coefficient of the interaction term, only when *Con*_*is*_ and *COVID*_*ts*_ are both 1, *β*_1_ exists, that is, *β*_1_ reflects the effect that the interaction term is 1 and only 1. *COVID*_*ts*_ is whether COVID-19 occurred in year t in region s, it is our proxy variable to measure before and after the outbreak, and *β*_2_ is the coefficient of the proxy variable. *Con*_*is*_ is the proxy variable of whether there are confirmed cases of COVID-19 in the living area of residents in area s, and *β*_3_ is the coefficient of the proxy variable. *X*_*its*_ are exogenous variables that may be related to personal and family characteristics that affect test scores, including gender, age, ethnicity, family income, number of family members, whether they are sick, etc. *β*_4_ is the coefficient of these exogenous variables. *β*_0_ and *ε*_*its*_ represent the constant term and the error term, respectively.

### Empirical results

After the outbreak of COVID-19, some areas were detected to have COVID-19 infection, so these areas were implemented "static management" measures to prevent the spread of infection, other healthy residents in the area were in a state of "frightened", afraid Infected by the virus, so these residents will adjust their behavior to protect themselves from infection, they may temporarily reserve relevant knowledge or medicines to reduce the probability of infection, including knowledge and products related to traditional Chinese medicine, but for those who do not appear to be infected As far as the residents in the areas where they live, their daily life is not restricted by "static management", so most of them will not be in a state of "unfounded worries" because they believe in the management ability of the government, and they will not prepare in advance. In this way, the two types of residents show different coping states, so there will be differences in their TCM health literacy afterward.

#### Baseline results

We expect that in areas where there have been confirmed cases of COVID-19, residents living there will feel anxious because they are worried about being infected, which will cause certain necessary adjustments to their health behaviors, and the health literacy of these residents in traditional Chinese medicine will be improved the rise was higher compared to residents living in areas without a confirmed case of COVID-19. The estimated results are shown in Table 2, in which the first row estimates the impact of whether there is a confirmed case of COVID-19 in a resident’s territory on the residents’ TCM health literacy. Model (1)—Model (9) We add other variables that may affect the health literacy of traditional Chinese medicine to the equation and focus on whether there is a confirmed case and whether it is the result of the cross coefficient of the epidemic year, At the same time, we conducted a multicollinearity test for each model, and the test results were ideal (S3 Table).

**Table 2:** The impact of confirmed cases on the literacy of residents in traditional Chinese medicine.

Variable	Interpreted variable: Score								
	(1)	(2)	(3)	(4)	(5)	(6)	(7)	(8)	(9)
Con*COVID	3.458*** (1.145)	4.441*** (1.111)	4.482*** (1.115)	7.040*** (1.011)	6.949*** (1.009)	4.624*** (1.077)	4.857*** (1.078)	4.926*** (1.079)	4.882*** (1.079)
COVID	11.538*** (0.840)	9.897*** (0.848)	9.877*** (0.850)	10.869*** (0.722)	12.336*** (0.774)	13.516*** (0.793)	14.075*** (0.835)	13.861*** (0.843)	13.846*** (0.842)
Con	14.025*** (0.878)	12.118*** (0.873)	12.037*** (0.889)	6.737*** (0.819)	6.906*** (0.818)	7.842*** (0.831)	7.601*** (0.836)	7.519*** (0.840)	7.560*** (0.840)
income		0.000*** (0.000)	0.000*** (0.000)	0.000*** (0.000)	0.000*** (0.000)	0.000*** (0.000)	0.000*** (0.000)	0.000*** (0.000)	0.000*** (0.000)
Occupation			0.135 (0.176)	0.563*** (0.153)	0.477*** (0.153)	0.455*** (0.153)	0.468*** (0.153)	0.453*** (0.153)	0.461*** (0.153)
Education				6.324*** (0.199)	6.198*** (0.202)	5.957*** (0.204)	5.931*** (0.204)	6.025*** (0.211)	5.972*** (0.214)
Population					-0.933*** (0.164)	-0.628*** (0.168)	-0.601*** (0.168)	-0.538*** (0.171)	-0.545*** (0.170)
Han						6.505*** (0.857)	6.430*** (0.855)	6.337*** (0.853)	6.353*** (0.853)
Sick							-1.511** (0.592)	-1.256** (0.611)	-1.279** (0.611)
Age								0.042** (0.020)	0.039 (0.020)
Gender									-0.727 (0.498)
Constant	37.956*** (0.615)	35.122*** (0.694)	34.312*** (1.327)	18.592*** (1.170)	21.172*** (1.254)	14.784*** (1.503)	15.372*** (1.520)	13.117*** (1.874)	14.438*** (2.091)
Observation	4016	4016	4016	4016	4016	4016	4016	4016	4016
R-squared	0.246	0.289	0.289	0.432	0.435	0.443	0.445	0.445	0.445

Note: Robust standard errors in brackets, significant level

*p<0.10

**p<0.05

***p<0.01.

First, we report the underlying regression estimates for the overall sample data. It was found that comparing the residents with confirmed COVID-19 patients in their living areas and the residents without confirmed patients with COVID-19, the health literacy of traditional Chinese medicine in the former was significantly higher than that of the latter. In contrast, the level of Chinese medicine literacy of residents in areas with confirmed COVID-19 patients was 3.5–7 points higher than that of residents in areas without confirmed patients with COVID-19, and the level of TCM literacy was still significant at 1% after adding relevant variables that may affect health literacy. It is significant at the level and the estimated coefficient estimates are relatively stable. This estimation result strongly verifies our conjecture that, under other conditions being equal, if a resident has a confirmed case of COVID-19 in his living area, this external factor will greatly improve the resident’s traditional Chinese medicine health cultural literacy [42–44].

Second, we also estimated the different responses of residents with different educational levels when faced with confirmed cases of COVID-19 in their living areas, that is, the impact on TCM health literacy. Similarly, we also controlled for relevant variables that may affect TCM literacy, and the results are shown in Table 3. We found that for uneducated (illiterate) residents, whether there was a confirmed case of COVID-19 in their living area was positively, but not significantly, the improvement in medical and health literacy. This is consistent with the scientific literature on health inequality. Consistent with repeated evidence of a strong association between lower education levels and poorer health literacy outcomes [45–50]. For residents with secondary education (primary or secondary education) and higher education (university or above), if there is a confirmed case of COVID-19 in their living area, the improvement in their TCM health literacy level is Significant, the specific performance is as follows: for residents with secondary education, the TCM health literacy of residents in areas with confirmed COVID-19 cases is about 8 points higher than that in areas without confirmed cases of COVID-19, and at a significant level of 1%. Significantly, the coefficient is stable with or without the addition of control variables; for residents with higher education, the TCM health literacy of residents in areas with confirmed COVID-19 cases is about 6 points higher than that in areas without confirmed cases of COVID-19, and in It is significant at the 5% significance level, and the coefficient is stable with or without the addition of control variables.

**Table 3:** The impact of confirmed cases on the literacy of residents in traditional Chinese medicine (by education level).

Variable	Uneducated		Middle education		Higher education	
	(1)	(2)	(3)	(4)	(5)	(6)
Con*COVID	3.961 (2.427)	0.037 (3.112)	7.922*** (1.356)	8.191*** (1.402)	6.374** (2.555)	5.523** (2.530)
COVID	13.794*** (1.260)	17.176*** (1.639)	10.081*** (0.963)	12.006*** (1.104)	7.541*** (2.256)	7.987*** (2.630)
Con	11.995*** (2.054)	13.868*** (2.281)	8.506*** (1.058)	6.503*** (1.127)	1.383 (1.929)	0.832 (1.907)
Income		0.000*** (0.000)		0.000*** (0.000)		0.000*** (0.000)
Occupation		2.458** (1.026)		1.520*** (0.285)		-0.404** (0.201)
Population		-1.142*** (0.353)		-0.656*** (0.228)		-0.454 (0.506)
Han		6.636*** (1.675)		7.477*** (1.233)		1.778 (2.259)
Sick		-0.347 (1.122)		-1.378* (0.820)		1.046 (1.738)
Age		0.003 (0.047)		-0.000 (0.026)		0.094 (0.046)
Gender		-2.059* (1.089)		-0.488 (0.663)		-0.288 (1.063)
Constant	24.275*** (0.854)	6.701 (6.947)	42.705*** (0.699)	26.999*** (2.812)	59.447*** (1.727)	54.311*** (4.345)
Observation	996	996	2374	2374	646	646
R-squared	0.308	0.351	0.253	0.299	0.180	0.214

Note: The mediation between residents’ health literacy and their socioeconomic status lies in residents’ educational attainment, which is also of particular interest to us. We classify the surveyed residents according to their education level, and classify residents with an education level equal to 1 as "Uneducated"; classify residents with an education level greater than 1 and less than or equal to 4 as "Middle education"; Residents with a level greater than 4 were classified as "Higher education", with robust standard errors in parentheses, significant levels

*p<0.10

**p<0.05

***p<0.01.

Finally, we subdivide TCM health literacy. In the TCM health literacy test, we summed up the three test scores of TCM cultural knowledge, TCM basic concepts, and TCM information comprehension ability, and defined it as a "theoretical knowledge score"; Methods The sum of the scores for the two tests is defined as the "Approach Score". Its purpose is to explore the specific aspects of the improvement of residents’ TCM literacy. Is this improvement in theory or practice? The results are shown in Table 4. In terms of "theoretical knowledge score", the improvement of TCM health literacy of residents in areas without confirmed cases of COVID-19 was significantly higher than that of residents in areas with confirmed cases of COVID-19. Compared with the latter, the health literacy of traditional Chinese medicine is improved by about 3 points, which is significant at the significant level of 1%, and the coefficient of whether the control variable is added or not is stable; and for the "application method score", the traditional Chinese medicine of residents in areas with confirmed cases of COVID-19 The improvement of health literacy is significantly higher than that of residents in areas without confirmed cases of COVID-19. The specific manifestation is that the health literacy of traditional Chinese medicine in the former is improved by about 7 points compared with the latter, and it is significant at the 1% significant level. Whether to add a control variable The coefficients are stable.

**Table 4:** The impact of confirmed cases on the literacy of residents in traditional Chinese medicine (by subject).

Variable	Theoretical knowledge score		Application method score	
	(1)	(2)	(3)	(4)
Con*COVID	-3.407*** (0.644)	-2.526*** (0.601)	7.016*** (0.583)	7.571*** (0.571)
COVID	5.686*** (0.481)	6.823*** (0.473)	5.852*** (0.411)	7.030*** (0.436)
Con	7.231*** (0.492)	3.468*** (0.462)	6.794*** (0.439)	4.088*** (0.440)
Income		0.000*** (0.000)		0.000*** (0.000)
Occupation		0.207** (0.085)		0.250*** (0.083)
Education		3.487*** (0.119)		2.490*** (0.114)
Population		-0.208** (0.094)		-0.356*** (0.095)
Han		3.483*** (0.474)		2.826*** (0.459)
Sick		-0.807** (0.337)		-0.442 (0.325)
Age		0.013 (0.011)		0.026** (0.011)
Gender		-0.759*** (0.278)		0.023 (0.267)
Constant	16.593*** (0.352)	4.234*** (1.161)	21.362*** (0.299)	10.255*** (1.126)
Observation	4016	4016	4016	4016
R-squared	0.106	0.358	0.378	0.491

Note: This table is categorized by scoring subjects. To facilitate observation and discussion, we summed up the three test scores of Chinese medicine cultural knowledge, basic concepts of Chinese medicine, and Chinese medicine information comprehension ability in the Chinese medicine health literacy test, and defined it as a "Theoretical knowledge score"; The sum of the scores of the two tests of the method and the public appropriate method of traditional Chinese medicine is defined as the "Application method score". Robust standard errors in parentheses, significant levels

*p<0.10

**p<0.05

***p<0.01.

#### Placebo test

The key identification assumption of the DID method is that the emergence of confirmed cases of COVID-19 has no significant impact on residents’ health literacy of traditional Chinese medicine. To test the validity of this hypothesis, we set up two placebo tests. The first test is to randomly generate the experimental group. We randomly assign the values of the variable "COVID" and the variable "Con" to "0 or 1", respectively. The second test is to change the time of occurrence of COVID-19, and use the survey data from 2018–2019 (see S2 Table and S1 Appendix Description for the relevant information on the 2018 data) to replace the original data from 2019–2020 for basic regression.

**Fig 2:**
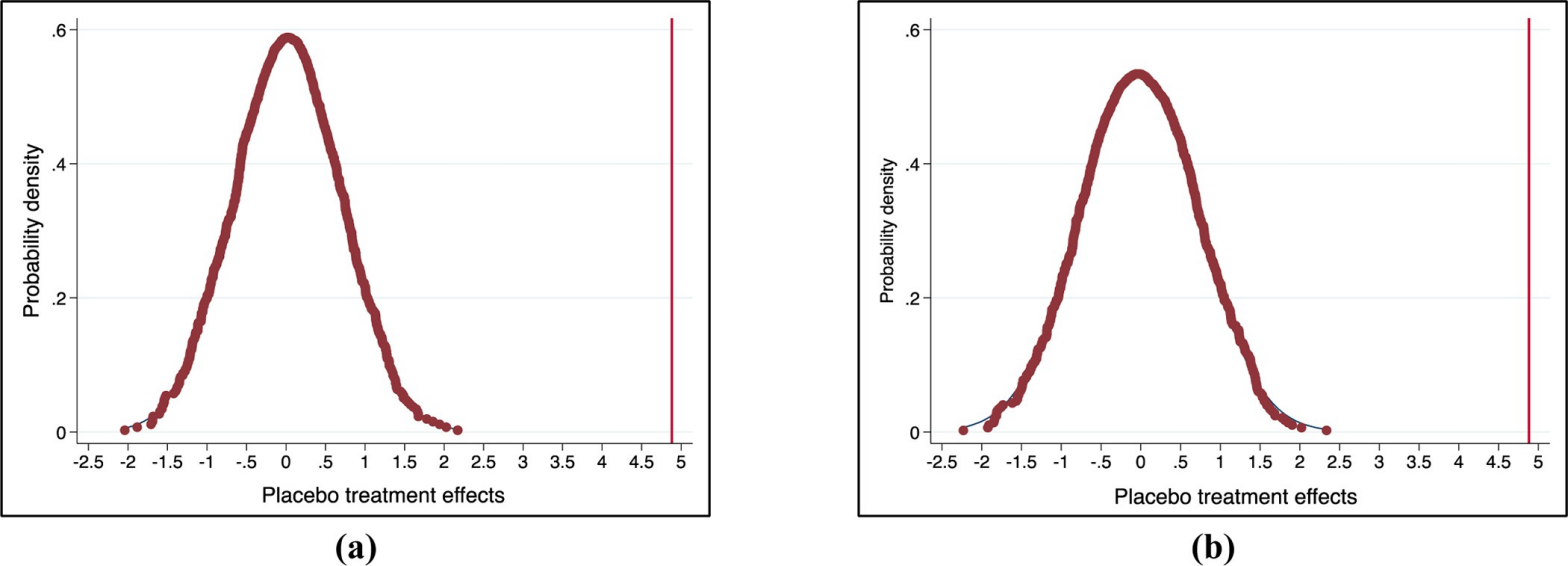
Placebo effect on the impact of confirmed cases on residents’ TCM literacy (replacement test). Note: This figure shows the estimated distribution of placebos using the randomly generated "COVID" variable and "Con" variable on the impact of residents’ TCM literacy, taking into account the time of the outbreak of COVID-19 and whether there are confirmed cases of COVID-19 in the region, which is randomly assigned, so we get each estimate by executing our main model specification (with the addition of control variables), and we do this process 1000 times. where a and b represent the random "COVID" variable and "Con" respectively.

In the test of the randomly generated experimental group, the null hypothesis is that the occurrence of confirmed cases of COVID-19 in the region does not affect residents’ TCM health literacy. Under the null hypothesis, the estimated coefficients from the actual data can be considered as random samples in the permutation distribution, we can generate permutation distributions of estimated coefficients and use them for statistical inference. On the one hand, we first randomly assign the occurrence period of COVID-19, and construct a placebo treatment status for each resident [51, 52], specifically, our database is 2019 and 2020 A total of two years of survey data, the judgment of the variable "COVID" is only 0 or 1 when randomly generating the experimental group, we will randomly assign the value of the "COVID" variable in the sample to "0 or 1", without repetition, use the randomly assigned "COVID" variable to combine with the original "Con" variable to form a new interaction term, and then make a new estimate. On the other hand, similar to the former, we just replace the randomly assigned variable with "Con", combine it with the original "COVID" variable to form a new interaction term, and then make a new estimate. Fig 2 shows the distribution of the placebo treatment effect from 1000 random assignments, the solid line shows the estimated treatment effect from the baseline analysis, placebo with an absolute value equal to or greater than the corresponding estimate from the baseline analysis when the P value of the permuted placebo test is replaced For the estimated proportion, we found that the P values were significant within the 1%-5% confidence interval in the test, so it rejected the invalid null hypothesis that the effect of the confirmed cases of COVID-19 in the region is not entirely due to endogeneity Bias, that is to say, there may be an endogenous bias in the effect of confirmed cases of COVID-19 in a region, but there is a great possibility that there will be an additional effect based on this endogenous bias. This effect cannot be zero, which will also provide support for our further identification strategy.

In our tests of changing the timing of COVID-19, we used a sample of survey data from 2018–2019. COVID only broke out in Gansu, China in early 2020. We use the TCM health culture of residents in Gansu Province in 2018 and assume that the epidemic broke out in Gansu in early 2019, and we expect the "placebo" treatment status to be insignificant. The data used for this test will not be affected by COVID-19. Using data from 2018 and 2019 has several advantages. First of all, the surveys in 2018 and 2019 were sample surveys conducted across the province before the outbreak of COVID-19, and the testing areas have not changed, making our testing more targeted. Secondly, the data included in 2018 is the same as the data indicators included in 2019 and 2020, which allows us to use the model specifications shown in Table 2 to estimate the impact of confirmed cases of COVID-19 in the region on the health of residents. The impact of literacy. Finally, the two sample sizes we use ("2018–2019" and "2019–2020" samples) are close to the same, which can avoid problems such as inconsistency and inaccuracy in estimation caused by large gaps in sample sizes. The results of this placebo test are shown in Table 5. We did not find any statistically significant results in model (1)-model (9) interaction coefficients. Therefore, we reject the validity of the DID identification hypothesis, and there are sufficient reasons to believe that the emergence of confirmed cases of COVID-19 will significantly impact the health literacy of traditional Chinese medicine among local residents.

**Table 5:** The impact of confirmed cases on the literacy of residents in traditional Chinese medicine.

Variable	Interpreted variable: Score								
	(1)	(2)	(3)	(4)	(5)	(6)	(7)	(8)	(9)
Con*COVID	0.647 (1.221)	-0.222 (1.207)	-0.205 (1.206)	-1.156 (1.104)	-0.093 (1.099)	-0.227 (1.107)	-0.204 (1.110)	-0.204 (1.110)	-0.189 (1.111)
COVID	-2.491*** (0.846)	-2.357*** (0.830)	-2.344*** (0.830)	-3.112*** (0.743)	-5.031*** (0.787)	-5.034*** (0.787)	-5.065*** (0.789)	-5.063*** (0.792)	-5.102*** (0.796)
Con	13.378*** (0.849)	12.766*** (0.848)	12.554*** (0.862)	7.893*** (0.824)	6.900*** (0.820)	6.869*** (0.822)	6.865*** (0.823)	6.863*** (0.825)	6.823*** (0.829)
Income		0.000*** (0.000)	0.000*** (0.000)	0.000*** (0.000)	0.000*** (0.000)	0.000*** (0.000)	0.000*** (0.000)	0.000*** (0.000)	0.000*** (0.000)
Occupation			0.320 (0.196)	0.611*** (0.172)	0.576*** (0.172)	0.576*** (0.171)	0.575*** (0.171)	0.575*** (0.171)	0.568*** (0.172)
Education				6.351*** (0.227)	6.341*** (0.227)	6.364*** (0.228)	6.381*** (0.232)	6.383*** (0.236)	6.410*** (0.238)
Population					-1.238*** (0.225)	-1.242*** (0.225)	-1.237*** (0.226)	-1.236*** (0.227)	-1.233*** (0.227)
Han						-1.276 (1.517)	-1.292 (1.517)	-1.292 (1.518)	-1.310 (1.517)
Sick							0.232 (0.658)	0.229 (0.660)	0.228 (0.660)
Age								0.001 (0.022)	0.002 (0.022)
Gender									0.377 (0.554)
Constant	40.447*** (0.581)	38.113*** (0.667)	36.168*** (1.397)	21.431*** (1.308)	25.997*** (1.504)	27.217*** (2.081)	27.131*** (2.097)	27.076*** (2.428)	26.477*** (2.584)
Observation	3,889	3889	3889	3889	3889	3889	3889	3889	3889
R-squared	0.117	0.147	0.148	0.291	0.296	0.297	0.297	0.297	0.297

Note: Robust standard errors in brackets, significant level

*p<0.10

**p<0.05

***p<0.01.

#### Mechanism

In addition to the main results, we further discussed the reasons why the presence of confirmed COVID-19 cases in the region would promote the improvement of TCM literacy among residents.

First, we defined the ways or mechanisms for improving residents’ TCM health literacy. It is believed that the main source of residents’ acquisition of TCM health quality is learning TCM knowledge, so what mechanism residents use to obtain TCM knowledge is the key topic discussed in this part. In our survey, there are various ways for residents to acquire knowledge of Chinese medicine, such as publicity boards, mass media, printed materials, video materials, TCM free clinics, theme activities, theme lectures, etc. To facilitate the discussion, we classify these ways, that is, the traditional methods of residents through propaganda boards, mass media, printed materials, video materials, etc. are classified as "the first type of mechanism". This mechanism generally exists in public life. And it is the main method of health literacy knowledge in China, which is characterized by a one-way acquisition mechanism of "people to things". Residents are classified as "second-type mechanisms" through special methods such as TCM-free clinics, theme activities, and theme lectures. Partially existing in public life, it is a secondary method of health knowledge propaganda in China. It is characterized by a two-way acquisition mechanism of "people to people" in addition to the one-way communication mechanism of "people to objects". We allow residents to acquire knowledge of traditional Chinese medicine through these two types of mechanisms at the same time.

Second, we divide the total sample into two sub-samples according to different mechanisms for separate regression. In the total sample, 3159 residents obtained knowledge of traditional Chinese medicine through the first type of mechanism, and 2561 residents obtained knowledge of traditional Chinese medicine through the second type of mechanism. In this section, we discuss why residents in areas with confirmed cases have a greater improvement in TCM health literacy than residents in areas without confirmed cases. Are there different ways of acquiring knowledge of Chinese medicine between the former and the latter? With these problems in mind, we continued to use the estimation model of Eq (1) and regressed the two subsamples. The result of the regression indicated that (under this mechanism) the residents of areas with confirmed cases were more likely than the residents of areas without confirmed cases. Comparing the two sub-samples for the improvement of medical and health literacy, the larger the coefficient, the more preferred the residents in the areas with confirmed cases have for this type of mechanism, and the greater the improvement of the residents’ traditional Chinese medicine literacy by this mechanism. We expect that residents in areas with confirmed cases are more inclined to acquire knowledge of traditional Chinese medicine through the "second type of mechanism", and believe that the "second type of mechanism" can greatly improve the residents’ TCM health literacy.

Finally, the findings confirmed our expectations. As shown in Table 6, both types of mechanisms can have a significant positive impact on the improvement of residents’ traditional Chinese medicine literacy, which is shown in the following: For residents who have acquired traditional Chinese medicine health literacy through the first type of mechanism, areas with confirmed cases of COVID-19 Compared with residents in areas without confirmed cases of COVID-19, the TCM literacy of residents improved by 4.6%, which was significant at the 1% level, and the coefficient remained stable after adding control variables; for residents who obtained TCM health literacy through the first type of mechanism, The TCM literacy of residents in areas with confirmed cases of COVID-19 increased by 6%-7.3% compared with residents in areas without confirmed cases of COVID-19, which was significant at the 1% level, and the coefficient remained stable after adding control variables.

**Table 6:** The impact of confirmed cases on the literacy of residents in traditional Chinese medicine (by impact mechanism).

Variable	Type I mechanism		Type II mechanism	
	(1)	(2)	(3)	(4)
Con*COVID	4.573*** (1.202)	4.556*** (1.145)	6.091*** (1.311)	7.317*** (1.261)
COVID	9.600*** (0.902)	13.577*** (0.937)	9.837*** (1.025)	11.661*** (1.076)
Con	10.341*** (0.931)	6.590*** (0.909)	12.723*** (0.901)	7.941*** (0.900)
Income		0.000*** (0.000)		0.000*** (0.000)
Occupation		0.228 (0.154)		0.107 (0.171)
Education		4.802*** (0.246)		4.794*** (0.251)
Population		-0.645*** (0.186)		-0.518** (0.219)
Han		6.368*** (0.942)		5.239*** (1.076)
Sick		-1.295* (0.670)		-0.360 (0.732)
Age		0.046** (0.022)		0.053** (0.024)
Gender		-0.121 (0.539)		-0.236 (0.598)
Constant	43.403*** (0.672)	20.471*** (2.385)	41.715*** (0.662)	21.293*** (2.503)
Observation	3159	3159	2561	2561
R-squared	0.218	0.372	0.269	0.407

Note: This table shows the mechanism by which the occurrence of confirmed cases of COVID-19 in the region improves the literacy of residents in traditional Chinese medicine. Robust standard errors in parentheses, significance levels

*p<0.10

**p<0.05

***p<0.01.

## Conclusion

Using data from the TCM health literacy monitoring survey of residents in China (Gansu Province) in 2019 and 2020, we focused on the impact of confirmed cases on TCM health literacy. Thanks to the management and control policies of the central and local governments on the COVID-19 pandemic, the first round of the epidemic in Gansu Province can be effectively controlled, allowing us to have a perfect experimental sample and opportunities for in-depth research. The research is completely justified Consider the COVID-19 event as a powerful exogenous shock, and the emergence of confirmed cases of COVID-19 is an important manifestation of the exogenous shock.

By comparing the improvement gap in traditional Chinese medicine health literacy between residents in areas with confirmed cases of COVID-19 and those in areas without confirmed cases of COVID-19. We came to three main conclusions:

First, the occurrence of confirmed cases of COVID-19 will lead to a 3.5%-7.0% improvement in residents’ TCM health literacy. This is the original intention of our research and the basic conclusion of our research. This conclusion proves that confirmed cases of COVID-19 have a positive impact on residents’ TCM health literacy, indicating that after experiencing regional "static management", residents will adjust their health behaviors because of fear of being infected, and these behaviors may be related to TCM At this time, the residents’ TCM health literacy will be improved due to their behavior adjustment.

Second, the occurrence of confirmed cases of COVID-19 does not significantly improve the TCM health literacy of uneducated residents, but it will lead to an increase of about 8% and 6% in the TCM health literacy of residents with secondary education and higher education. "Static management" has no significant impact on the health literacy of traditional Chinese medicine among uneducated residents. This group of residents will only obey the government or other residents’ admonitions, and "staying at home" may be their only health behavior adjustment. "They are illiterate" may be the strongest explanation for this result. The occurrence of confirmed cases of COVID-19 has a greater impact on the TCM health literacy of secondary education residents than higher education residents. The reason is that the TCM health literacy of higher education residents is higher than that of secondary education residents. The upper limit is higher education residents have less room for improvement, and secondary education residents have more room for improvement.

Third, the occurrence of confirmed cases of COVID-19 affects residents’ TCM health literacy, which is mainly reflected in practical health literacy. Under the condition that all residents have the same time to acquire health knowledge of traditional Chinese medicine, the occurrence of confirmed cases of COVID-19 will make residents spend more time to acquire acquiring a practical knowledge of traditional Chinese medicine, and at this time, the time for residents to acquire theoretical knowledge of traditional Chinese medicine will be reduced. The resulting results were reflected in the residents’ tests: the presence of a confirmed case of COVID-19 resulted in a significant decrease in the resident’s "Theoretical Knowledge Score" and a significant increase in the "Approach Score". This kind of practical thinking corroborates the old saying circulated among Chinese residents, that "regardless of whether the cat is black or white, the cat that can catch mice is a good cat".

In addition to the main conclusions, we further explored how the emergence of confirmed cases of COVID-19 affects residents’ health behavior adjustment through heterogeneity analysis, and ultimately affects residents’ TCM health literacy, that is, residents’ preference for access to TCM knowledge. In the above, we believe that there are two ways to acquire knowledge of traditional Chinese medicine, and these two ways should have different degrees of influence on the health literacy of traditional Chinese medicine. Positively, our research also confirms this. If the knowledge of traditional Chinese medicine is only obtained through the "first type mechanism", then the occurrence of confirmed cases of COVID-19 will increase the residents’ traditional Chinese medicine health literacy by about 4.5%. %-7% or so. Therefore, after experiencing "static management", residents will prefer to acquire knowledge of traditional Chinese medicine through the "second type of mechanism". After the boring isolation is over, residents will have a strong need to release their depressed hearts, so they will prefer to choose interesting and active ways to acquire knowledge of traditional Chinese medicine. The "second type of mechanism" is exactly this way.

Although this study explores the impact of confirmed cases of COVID-19 on residents’ TCM health literacy from local data, it provides new evidence for the improvement of TCM health literacy among Chinese residents (during the COVID-19 pandemic). That is, during the COVID-19 pandemic, the emergence of confirmed cases will improve residents’ TCM health literacy. With the improvement of the Chinese medicine literacy of residents, it is beneficial to both society and individuals. In the short term, improved health literacy can help individuals reduce their risk of contracting COVID-19 and reduce the number of confirmed cases of COVID-19 in society. In the long run, the improvement of health literacy can improve the efficiency of residents’ medical treatment, protect the life and health of residents to a greater extent, promote the rational allocation of social medical resources, and reduce the waste of medical resources.

Reviewing our research, we think that research has some limitations. On the one hand, our sample size only covers the entire Gansu Province, and the situation in other regions may not be the same as in Gansu Province (the economy, culture, and politics of other regions may be different, but it will also affect the score), if it represents all regions of China, we think it is far-fetched. We did not collect data from other provinces, which is a pity that the research is limited and researchers cannot consider as many regions as possible. On the other hand, we have no way to objectively measure each person’s uniqueness, so there will be omissions in the variables we consider. This uniqueness may affect its score, such as personal understanding of Chinese medicine knowledge, preferences, etc. subjective inner consciousness. Since the data are cross-sectional, we cannot use individual fixed effects to reduce their bias in estimates.

## Supporting information

S1 Table Division of experimental and control groups. (DOCX)

S2 Table Summary statistics (by specific year). (DOCX)

S3 Table Multicollinearity tests for the main models. (DOCX)

S1 Appendix (DOCX)

## References

[1] Zarocostas John. "How to fight an infodemic." *The Lancet* 395.10225 (2020): 676. doi: 10.1016/S0140-6736(20)30461-X 32113495 PMC7133615

[2] Sørensen Kristine, et al. "Health literacy in Europe: comparative results of the European health literacy survey (HLS-EU)." *European journal of public health* 25.6 (2015): 1053–1058. doi: 10.1093/eurpub/ckv043 25843827 PMC4668324

[3] Nutbeam Don. "Discussion paper on promoting, measuring and implementing health literacy-implications for policy and practice in non-communicable disease prevention and control." *World Health Organ* (2017): 1–29.

[4] Paakkari L, Okan O. COVID-19: health literacy is an underestimated problem[J]. The Lancet Public Health, 2020, 5(5): e249–e250 32302535 10.1016/S2468-2667(20)30086-4 PMC7156243

[5] Scheid V. The globalisation of Chinese medicine[J]. The Lancet, 1999, 354: SIV10. 10.1016/s0140-6736(99)90353-7 10691420

[6] Harnett S, Morgan-Daniel J. Health literacy considerations for users of complementary and alternative medicine[J]. Journal of Consumer Health on the Internet, 2018, 22(1): 63–71.

[7] Yu X, Li N. How did Chinese government implement unconventional measures against COVID-19 pneumonia[J]. Risk management and healthcare policy, 2020, 13: 491. 32581611 10.2147/RMHP.S251351 PMC7266822

[8] Qian D, Li O. The relationship between risk event involvement and risk perception during the COVID‐19 outbreak in China[J]. Applied Psychology: Health and Well‐Being, 2020, 12(4): 983–999. doi: 10.1111/aphw.12219 32829535 PMC7461204

[9] He X, Zhou C, Wang Y, et al. Risk assessment and prediction of COVID-19 based on epidemiological data from spatiotemporal geography[J]. Frontiers in Environmental Science, 2021, 9: 634156.

[10] Stormacq C, Van den Broucke S, Wosinski J. Does health literacy mediate the relationship between socioeconomic status and health disparities? Integrative review[J]. Health promotion international, 2019, 34(5): e1–e17. 30107564 10.1093/heapro/day062

[11] Baker D W. The meaning and the measure of health literacy[J]. Journal of general internal medicine, 2006, 21(8): 878–883. 16881951 10.1111/j.1525-1497.2006.00540.x PMC1831571

[12] Nutbeam D. The evolving concept of health literacy[J]. Social science & medicine, 2008, 67(12): 2072–2078. 18952344 10.1016/j.socscimed.2008.09.050

[13] Kickbusch I S. Health literacy: addressing the health and education divide[J]. Health promotion international, 2001, 16(3): 289–297 11509466 10.1093/heapro/16.3.289

[14] Sørensen K, Van den Broucke S, Fullam J, et al. Health literacy and public health: a systematic review and integration of definitions and models[J]. BMC public health, 2012, 12(1): 1–13. 22276600 10.1186/1471-2458-12-80 PMC3292515

[15] Nutbeam D. Health literacy as a public health goal: a challenge for contemporary health education and communication strategies into the 21st century[J]. Health promotion international, 2000, 15(3): 259–267.

[16] Zhang X, Luo W, Zhu J. Top-down and bottom-up lockdown: evidence from COVID-19 prevention and control in China[J]. Journal of Chinese Political Science, 2021, 26(1): 189–211. 33424220 10.1007/s11366-020-09711-6 PMC7784223

[17] Kraemer M U G, Yang C H, Gutierrez B, et al. The effect of human mobility and control measures on the COVID-19 epidemic in China[J]. Science, 2020, 368(6490): 493–497. 32213647 10.1126/science.abb4218 PMC7146642

[18] Hadjidemetriou G M, Sasidharan M, Kouyialis G, et al. The impact of government measures and human mobility trend on COVID-19 related deaths in the UK[J]. Transportation research interdisciplinary perspectives, 2020, 6: 100167. 34173458 10.1016/j.trip.2020.100167 PMC7334915

[19] Tian H, Liu Y, Li Y, et al. An investigation of transmission control measures during the first 50 days of the COVID-19 epidemic in China[J]. Science, 2020, 368(6491): 638–642. 32234804 10.1126/science.abb6105 PMC7164389

[20] Chudik A, Pesaran M H, Rebucci A. Voluntary and mandatory social distancing: Evidence on COVID-19 exposure rates from Chinese provinces and selected countries[R]. National Bureau of Economic Research, 2020.

[21] Brown C S, Ravallion M. Inequality and the coronavirus: Socioeconomic covariates of behavioral responses and viral outcomes across US counties[R]. national Bureau of economic research, 2020.

[22] Brienen N C J, Timen A, Wallinga J, et al. The effect of mask use on the spread of influenza during a pandemic[J]. Risk Analysis: An International Journal, 2010, 30(8): 1210–1218. 10.1111/j.1539-6924.2010.01428.x PMC7169241 20497389

[23] Duan T, Jiang H, Deng X, et al. Government intervention, risk perception, and the adoption of protective action recommendations: evidence from the COVID-19 prevention and control experience of China[J]. International Journal of Environmental Research and Public Health, 2020, 17(10): 3387. 32414013 10.3390/ijerph17103387 PMC7277925

[24] Wang B, Liu J, Li Y, et al. Airborne particulate matter, population mobility and COVID-19: a multi-city study in China[J]. BMC public health, 2020, 20(1): 1–10. 33087097 10.1186/s12889-020-09669-3 PMC7576551

[25] Jiang J, Luo L. Influence of population mobility on the novel coronavirus disease (COVID-19) epidemic: based on panel data from Hubei, China[J]. Global Health Research and Policy, 2020, 5(1): 1–10. 32518832 10.1186/s41256-020-00151-6 PMC7276249

[26] Liu K, Ai S, Song S, et al. Population movement, city closure in Wuhan, and geographical expansion of the COVID-19 infection in China in January 2020[J]. Clinical infectious diseases, 2020, 71(16): 2045–2051. 32302377 10.1093/cid/ciaa422 PMC7188136

[27] Pincombe M, Reese V, Dolan C B. The effectiveness of national-level containment and closure policies across income levels during the COVID-19 pandemic: an analysis of 113 countries[J]. Health Policy and Planning, 2021, 36(7): 1152–1162. 33942081 10.1093/heapol/czab054 PMC8135717

[28] Emeto T I, Alele F O, Ilesanmi O S. Evaluation of the effect of border closure on COVID-19 incidence rates across nine African countries: an interrupted time series study[J]. Transactions of The Royal Society of Tropical Medicine and Hygiene, 2021, 115(10): 1174–1183. 33690835 10.1093/trstmh/trab033 PMC7989183

[29] Bendavid E, Oh C, Bhattacharya J, et al. Assessing mandatory stay-at-home and business closure effects on the spread of COVID-19[J]. European journal of clinical investigation, 2021, 51(4): e13484. 33400268 10.1111/eci.13484 PMC7883103

[30] Wei J, Wang F, Zhao D. A risk perception model: Simulating public response to news reports in China[J]. Information Research, 2012, 17(2): 17–2.

[31] Prati G, Pietrantoni L, Zani B. A social‐cognitive model of pandemic influenza H1N1 risk perception and recommended behaviors in Italy[J]. Risk Analysis: An International Journal, 2011, 31(4): 645–656. 10.1111/j.1539-6924.2010.01529.x 21077927

[32] Lindell M K, Hwang S N. Households’ perceived personal risk and responses in a multihazard environment[J]. Risk Analysis: An International Journal, 2008, 28(2): 539–556. 10.1111/j.1539-6924.2008.01032.x 18419668

[33] Wang F, Wei J, Shi X. Compliance with recommended protective actions during an H7N9 emergency: a risk perception perspective[J]. Disasters, 2018, 42(2): 207–232. 28799670 10.1111/disa.12240

[34] Kasperson R E, Renn O, Slovic P, et al. The social amplification of risk: A conceptual framework[J]. Risk analysis, 1988, 8(2): 177–187.

[35] Slovic P. Perception of risk[J]. Science, 1987, 236(4799): 280–285. 3563507 10.1126/science.3563507

[36] Cori L, Bianchi F, Cadum E, et al. Risk perception and COVID-19[J]. International journal of environmental research and public health, 2020, 17(9): 3114. 32365710 10.3390/ijerph17093114 PMC7246460

[37] Wise T, Zbozinek T D, Michelini G, et al. Changes in risk perception and self-reported protective behaviour during the first week of the COVID-19 pandemic in the United States[J]. Royal Society open science, 2020, 7(9): 200742. 33047037 10.1098/rsos.200742 PMC7540790

[38] Xie K, Liang B, Dulebenets M A, et al. The impact of risk perception on social distancing during the COVID-19 pandemic in China[J]. International journal of environmental research and public health, 2020, 17(17): 6256. 32867381 10.3390/ijerph17176256 PMC7503995

[39] He S, Chen S, Kong L, et al. Analysis of risk perceptions and related factors concerning COVID-19 epidemic in Chongqing, China[J]. Journal of Community Health, 2021, 46(2): 278–285. 32592160 10.1007/s10900-020-00870-4 PMC7318903

[40] Kiel K A, McClain K T. House prices during siting decision stages: The case of an incinerator from rumor through operation[J]. Journal of Environmental Economics and Management, 1995, 28(2): 241–255.

[41] Chen Y, Fan Z, Gu X, et al. Arrival of young talent: The send-down movement and rural education in China[J]. American Economic Review, 2020, 110(11): 3393–3430.

[42] Silva M J, Santos P. The impact of health literacy on knowledge and attitudes towards preventive strategies against COVID-19: a cross-sectional study[J]. International journal of environmental research and public health, 2021, 18(10): 5421. 34069438 10.3390/ijerph18105421 PMC8159089

[43] Feinberg I. Building a culture of health literacy during COVID‐19[J]. New Horizons in Adult Education and Human Resource Development, 2021, 33(2): 60.

[44] Lüdecke D, Von Dem Knesebeck O. Protective behavior in course of the COVID-19 outbreak—survey results from Germany[J]. Frontiers in public health, 2020, 8: 572561. 33072712 10.3389/fpubh.2020.572561 PMC7543680

[45] Van Der Heide I, Wang J, Droomers M, et al. The relationship between health, education, and health literacy: results from the Dutch Adult Literacy and Life Skills Survey[J]. Journal of health communication, 2013, 18(sup1): 172–184. 24093354 10.1080/10810730.2013.825668 PMC3814618

[46] Kunst Anton E., et al. "Trends in socioeconomic inequalities in self-assessed health in 10 European countries." International Journal of Epidemiology 34.2 (2005): 295–305. doi: 10.1093/ije/dyh342 15563586

[47] Mackenbach Johan P., et al. "Socioeconomic inequalities in health in 22 European countries." New England journal of medicine 358.23 (2008): 2468–2481. doi: 10.1056/NEJMsa0707519 18525043

[48] Cutler, David M., and Adriana Lleras-Muney. "Education and health: insights from international comparisons." (2012).

[49] Lee Shoou-Yih D., et al. "Health literacy, health status, and healthcare utilization of Taiwanese adults: results from a national survey." BMC public health 10.1 (2010): 1–8. doi: 10.1186/1471-2458-10-614 20950479 PMC2967535

[50] Wittink H, Oosterhaven J. Patient education and health literacy[J]. Musculoskeletal Science and Practice, 2018, 38: 120–127. 30017902 10.1016/j.msksp.2018.06.004

[51] Lu F, Anderson M L. Peer effects in microenvironments: The benefits of homogeneous classroom groups[J]. Journal of Labor Economics, 2015, 33(1): 91–122.

[52] Rosenbaum P R. Interference between units in randomized experiments[J]. Journal of the American statistical association, 2007, 102(477): 191–200.

