# Supplementary material for: The impact of confirmed cases of COVID-19 on residents’ traditional Chinese medicine health literacy: A survey from Gansu Province of China
Source: PLoS One. 2023 Nov 14;18(11):e0285744. doi: 10.1371/journal.pone.0285744 (PMC10645358; doi:10.1371/journal.pone.0285744)
Supplement: S1 Table — (DOCX) [file pone.0285744.s001.docx]

**S1 Table. Division of experimental and control groups**

| **Year** | **“Con” value** | **Area** |
| --- | --- | --- |
| 2018 | 1 | Chengguan District, Huining County, Jingning County, Qinzhou District |
|  | 0 | Jingyuan County, Shiwen County, Huan County, Liangzhou District |
|  | | |
| 2019 | 1 | Chengguan District, Huining County, Min County, Kongtong District |
|  | 0 | Guanghe County, Huan County, Linze County, Pingchuan District |
|  | | |
| 2020 | 1 | Chengguan District, Kongtong District, Lingtai County, Qin'an County |
|  | 0 | Dangchang County, Pingchuan District, Shandan County, Zhenyuan County |

Note: The "Con" value is determined according to whether there are confirmed cases of COVID-19 in the area where the respondent is located. The data of the confirmed cases comes from the Gansu Provincial Health Commission.
