# Supplementary material for: The impact of confirmed cases of COVID-19 on residents’ traditional Chinese medicine health literacy: A survey from Gansu Province of China
Source: PLoS One. 2023 Nov 14;18(11):e0285744. doi: 10.1371/journal.pone.0285744 (PMC10645358; doi:10.1371/journal.pone.0285744)
Supplement: S2 Table — (DOCX) [file pone.0285744.s002.docx]

**A2 Table. Summary statistics (by specific year)**

| **Year** | **Variable** | **Obs.** | **Mean** | **Std. dev.** | **Min** | **Max** |
| --- | --- | --- | --- | --- | --- | --- |
| 2018 | Score | 1920 | 47.16458 | 19.77977 | 0 | 96 |
|  | Age | 1920 | 48.41719 | 11.94164 | 15 | 69 |
|  | Income | 1920 | 28854.65 | 37652.01 | 3 | 500001 |
|  | Population | 1920 | 3.139583 | 1.591829 | 1 | 11 |
|  | | | | | | |
| 2019 | Score | 1969 | 44.85119 | 20.70335 | 2 | 92 |
|  | Age | 1969 | 47.63281 | 12.90698 | 15 | 69 |
|  | Income | 1969 | 31994.29 | 40472.88 | 0 | 800000 |
|  | Population | 1969 | 2.026917 | 0.870582 | 1 | 5 |
|  | | | | | | |
| 2020 | Score | 2047 | 58.12115 | 18.82826 | 2 | 96 |
|  | Age | 2047 | 46.97411 | 13.41979 | 15 | 69 |
|  | Income | 2047 | 41833.10 | 33836.41 | 2000 | 300000 |
|  | Population | 2047 | 3.627259 | 1.791503 | 1 | 16 |

Note: Different from the summary statistics in the main text (Table 1), this table does not explain variables and does not show all categorical variables.
