# Supplementary material for: The impact of confirmed cases of COVID-19 on residents’ traditional Chinese medicine health literacy: A survey from Gansu Province of China
Source: PLoS One. 2023 Nov 14;18(11):e0285744. doi: 10.1371/journal.pone.0285744 (PMC10645358; doi:10.1371/journal.pone.0285744)
Supplement: S3 Table — (DOCX) [file pone.0285744.s003.docx]

**A3 Table. Multicollinearity tests for the main models**

| **Model** | **Table2** | **Table3** | **Table4** | **Table6** |
| --- | --- | --- | --- | --- |
| （1） | 2.34 | 2.37 | 2.34 | 2.47 |
| （2） | 2.04 | 1.92 | 1.67 | 1.68 |
| （3） | 1.85 | 2.32 | 2.34 | 2.21 |
| （4） | 1.80 | 1.66 | 1.67 | 1.60 |
| （5） | 1.79 | 3.54 | --- | --- |
| （6） | 1.79 | 2.26 | --- | --- |
| （7） | 1.77 | --- | --- | --- |
| （8） | 1.73 | --- | --- | --- |
| （9） | 1.67 | --- | --- | --- |
| Pass or not | Pass | Pass | Pass | Pass |

Note: The above data are all regression models, followed by multi-collinearity test. Since the multicollinearity test mainly verifies the accuracy of its main coefficients, we generally care about whether the coefficients are significant in Table 5, and we don’t care about the specific values of the coefficients, so we did not perform the multicollinearity test in the placebo test, because it is not necessary.
