# Supplementary material for: The impact of confirmed cases of COVID-19 on residents’ traditional Chinese medicine health literacy: A survey from Gansu Province of China
Source: PLoS One. 2023 Nov 14;18(11):e0285744. doi: 10.1371/journal.pone.0285744 (PMC10645358; doi:10.1371/journal.pone.0285744)
Supplement: S1 Appendix — (DOCX) [file pone.0285744.s004.docx]

**Appendix**

**About survey and data**

The objects of the survey on health literacy of traditional Chinese medicine in Gansu Province are the non-collective permanent residents aged 15-69 in 8 counties (cities, districts) of Gansu Province (residents who have lived in the local area for more than 6 months in the past 12 months, regardless of whether they have a local household registration). A multi-stage sampling method was used. In the first stage, 3 streets/towns were randomly selected in each county (city, district) in Gansu Province using probability proportional to size sampling (PPS); in the second stage, the PPS method was used to select 2 neighborhood committees/villages were selected from streets/towns; in the third stage, 60 households were randomly selected from the selected neighborhood committees/villages by simple random sampling; The 69-year-old resident population is the object of the survey, and each neighborhood committee/village plans to complete 45 questionnaires.

The questionnaire adopts the "Questionnaire on Chinese Citizens' Health and Cultural Literacy of Traditional Chinese Medicine" compiled by the Beijing University of Chinese Medicine. The questionnaire is mainly composed of informed consent, traditional Chinese medicine health culture literacy test questions (37 questions in total), basic information about the survey subjects, and a registration form for family members aged 15 to 69. The survey began in 2017 and is carried out once a year. The survey selects eight districts and counties in Gansu Province, monitors the health and cultural literacy of traditional Chinese medicine among the residents of these eight districts and counties, and collects relevant monitoring data (the counties and districts selected each year are different, So the respondents will also be different). In this study, we collected the survey data in 2018, 2019, and 2020. Among them, the survey monitoring and data collation in 2018 and 2019 were relatively smooth, and the data in 2019 showed the first confirmed case in Gansu Province the survey and monitoring work in 2020 was carried out after "zero confirmed cases" of COVID-19 in Gansu Province, and the survey feedback and data will be completed before 2021. This provides a suitable external experiment for us to study the impact of COVID-19 on the health literacy level of Chinese medicine among residents.

Because the selected survey sites are different each year, we have made great efforts to determine the samples of the experimental group and the control group, but this only represents the initial work of this study, so we did not focus on it in the main text. Regarding the determination of the value of the variable "Con", we mainly refer to the notifications of confirmed cases of COVID-19 from the Gansu Provincial Health and Health Commission from January 25 to February 17, 2020, and combine them with the investigation area to make One respondent's "Con" value was assigned (S1 Table).

**S1 Table. Division of experimental and control groups**

| **Year** | **“Con” value** | **Area** |
| --- | --- | --- |
| 2018 | 1 | Chengguan District, Huining County, Jingning County, Qinzhou District |
|  | 0 | Jingyuan County, Shiwen County, Huan County, Liangzhou District |
|  | | |
| 2019 | 1 | Chengguan District, Huining County, Min County, Kongtong District |
|  | 0 | Guanghe County, Huan County, Linze County, Pingchuan District |
|  | | |
| 2020 | 1 | Chengguan District, Kongtong District, Lingtai County, Qin'an County |
|  | 0 | Dangchang County, Pingchuan District, Shandan County, Zhenyuan County |

Note: The "Con" value is determined according to whether there are confirmed cases of COVID-19 in the area where the respondent is located. The data of the confirmed cases comes from the Gansu Provincial Health Commission.

The following is the three-year TCM health literacy monitoring data used in this study. The data set from 2019-2020 is mainly used for important research, and the data from 2018 is used for auxiliary research, that is, placebo test.

**S2 Table. Summary statistics (by specific year)**

| **Year** | **Variable** | **Obs.** | **Mean** | **Std. dev.** | **Min** | **Max** |
| --- | --- | --- | --- | --- | --- | --- |
| 2018 | Score | 1920 | 47.16458 | 19.77977 | 0 | 96 |
|  | Age | 1920 | 48.41719 | 11.94164 | 15 | 69 |
|  | Income | 1920 | 28854.65 | 37652.01 | 3 | 500001 |
|  | Population | 1920 | 3.139583 | 1.591829 | 1 | 11 |
|  | | | | | | |
| 2019 | Score | 1969 | 44.85119 | 20.70335 | 2 | 92 |
|  | Age | 1969 | 47.63281 | 12.90698 | 15 | 69 |
|  | Income | 1969 | 31994.29 | 40472.88 | 0 | 800000 |
|  | Population | 1969 | 2.026917 | 0.870582 | 1 | 5 |
|  | | | | | | |
| 2020 | Score | 2047 | 58.12115 | 18.82826 | 2 | 96 |
|  | Age | 2047 | 46.97411 | 13.41979 | 15 | 69 |
|  | Income | 2047 | 41833.10 | 33836.41 | 2000 | 300000 |
|  | Population | 2047 | 3.627259 | 1.791503 | 1 | 16 |

Note: Different from the summary statistics in the main text (Table 1), this table does not explain variables and does not show all categorical variables.

**Multicollinearity test of the main model**

We have conducted a multicollinearity test on the models in Table 2, Table 3, Table 4 and Table 6 in the text. The test results are shown in the table below. The VIF values in the table are all far less than 10, so we believe that these models pass the Collinearity test, the accuracy of its regression coefficient is worthy of recognition.

**S3 Table. Multicollinearity tests for the main models**

| **Model** | **Table2** | **Table3** | **Table4** | **Table6** |
| --- | --- | --- | --- | --- |
| （1） | 2.34 | 2.37 | 2.34 | 2.47 |
| （2） | 2.04 | 1.92 | 1.67 | 1.68 |
| （3） | 1.85 | 2.32 | 2.34 | 2.21 |
| （4） | 1.80 | 1.66 | 1.67 | 1.60 |
| （5） | 1.79 | 3.54 | --- | --- |
| （6） | 1.79 | 2.26 | --- | --- |
| （7） | 1.77 | --- | --- | --- |
| （8） | 1.73 | --- | --- | --- |
| （9） | 1.67 | --- | --- | --- |
| Pass or not | Pass | Pass | Pass | Pass |

Note: The above data are all regression models, followed by multi-collinearity test. Since the multicollinearity test mainly verifies the accuracy of its main coefficients, we generally care about whether the coefficients are significant in Table 5, and we don’t care about the specific values of the coefficients, so we did not perform the multicollinearity test in the placebo test , because it is not necessary.
